# Ethnicity Is an Independent Determinant of Age-Specific PSA Level: Findings from a Multiethnic Asian Setting

**DOI:** 10.1371/journal.pone.0104917

**Published:** 2014-08-11

**Authors:** Jasmine Lim, Nirmala Bhoo-Pathy, Selvalingam Sothilingam, Rohan Malek, Murali Sundram, Badrul Hisham Bahadzor, Teng Aik Ong, Keng Lim Ng, Sivaprakasam Sivalingam, Azad Hassan Abdul Razack

**Affiliations:** 1 Department of Surgery, Faculty of Medicine, University of Malaya, Kuala Lumpur, Malaysia; 2 Julius Center University of Malaya, Faculty of Medicine, University of Malaya, Kuala Lumpur, Malaysia; 3 Department of Urology, Tuanku Mizan Military Hospital, Kuala Lumpur, Malaysia; 4 Department of Urology, Selayang Hospital, Selangor, Malaysia; 5 Department of Urology, Kuala Lumpur Hospital, Kuala Lumpur, Malaysia; 6 Department of Surgery, University Kebangsaan Malaysia Medical Center, Kuala Lumpur, Malaysia; 7 Centre for Kidney Disease Research, School of Medicine, University of Queensland, Translational Research Institute, Brisbane, Australia; Innsbruck Medical University, Austria

## Abstract

**Objectives:**

To study the baseline PSA profile and determine the factors influencing the PSA levels within a multiethnic Asian setting.

**Materials and Methods:**

We conducted a cross-sectional study of 1054 men with no clinical evidence of prostate cancer, prostate surgery or 5α-reductase inhibitor treatment of known prostate conditions. The serum PSA concentration of each subject was assayed. Potential factors associated with PSA level including age, ethnicity, height, weight, family history of prostate cancer, lower urinary tract voiding symptoms (LUTS), prostate volume and digital rectal examination (DRE) were evaluated using univariable and multivariable analysis.

**Results:**

There were 38 men (3.6%) found to have a PSA level above 4 ng/ml and 1016 (96.4%) with a healthy PSA (≤4 ng/ml). The median PSA level of Malay, Chinese and Indian men was 1.00 ng/ml, 1.16 ng/ml and 0.83 ng/ml, respectively. Indians had a relatively lower median PSA level and prostate volume than Malays and Chinese, who shared a comparable median PSA value across all 10-years age groups. The PSA density was fairly similar amongst all ethnicities. Further analysis showed that ethnicity, weight and prostate volume were independent factors associated with age specific PSA level in the multivariable analysis (*p*<0.05).

**Conclusion:**

These findings support the concept that the baseline PSA level varies between different ethnicities across all age groups. In addition to age and prostate volume, ethnicity may also need to be taken into account when investigating serum PSA concentrations in the multiethnic Asian population.

## Introduction

Prostate cancer screening is widely practiced amongst primary care physicians and specialists, particularly in affluent western countries such as the United States and Canada [1], [2]. Latest reports of the European Randomized Study of Screening for Prostate Cancer (ERSPC) and Prostate, Lung Colorectal and Ovarian (PLCO) Cancer Screening Trial indicate that the prostate-specific antigen (PSA) testing increased the rate of overdiagnosis of patients with indolent and nonaggressive forms of prostate cancer to an estimated 17% to 50% [3], [4]. This is a major concern as a man with cancer that would remain asymptomatic for the remainder of his life is unlikely to benefit from screening or treatment. Therefore, the U.S. Preventive Services Task Force currently recommended against PSA screening in asymptomatic man [5]. New methods that improve the sensitivity and specificity of serum PSA level are essential particularly in identifying men at risk of high grade, aggressive prostate cancer.

One of the promising, non-invasive approaches is the application of prostate cancer risk prediction model which incorporates other indications for biopsy, in addition to serum PSA level. Evidence from the Prostate Cancer Prevention Trial (PCPT), the European Randomized Study of Screening for Prostate Cancer derived Prostate Risk Indicator (SWOP-PRI) and the Montreal cohort studies demonstrated that variables including age, ethnicity, family history of prostate cancer, prostate volume and digital rectal examination (DRE) could improve the positive predictive value of PSA [6]–[8].

With insights from western population-based studies, it is of our interest to study and improve the understanding of PSA profiles amongst Asian men. Malaysia is a high middle-income country in Southeast Asia with a multi-ethnic population encompassing mainly Malays, Chinese, Indians and indigenous races. We conducted a cross-sectional population-based study in the Klang Valley region, Malaysia to assess the distribution of PSA level across different age as well as ethnic groups and determine the association between age, ethnicity, family history of prostate cancer, height, weight, lower urinary tract voiding symptoms (LUTS), DRE and prostate volume on elevated PSA levels.

## Materials and Methods

### Study subjects and samples collection

The study was performed as part of the prostate awareness campaign held around the Klang Valley, Malaysia in July 2011. Men above 40 years of age were invited to eight participating hospitals comprising Kuala Lumpur Hospital, Selayang Hospital, Serdang Hospital, Sungai Buloh Hospital, Tengku Ampuan Rahimah Hospital, Tuanku Mizan Military Hospital, University Kebangsaan Malaysia Medical Centre and University Malaya Medical Centre. All hospitals except Serdang Hospital, Sungai Buloh Hospital and Tengku Ampuan Rahimah Hospital serve as the tertiary referral centers for urology services in the Klang Valley region. All participants provided written informed consent and ethical approval was granted from the medical research and ethics committee at the University Malaya Medical Centre and Malaysian Ministry of Health. A total of 1202 men participated in the campaign, consisting of Malay (423, 35.2%), Chinese (612; 50.9%), Indian (159; 13.2%) and ‘other ethnicities’ (8; 0.7%). Of these, only subjects with no history of prostate cancer, previous history of prostate surgery or 5α-reductase inhibitor treatment of known prostate conditions were selected for further analysis (N = 1160). All of them were aged between 40 and 79 years. Men from the ‘other ethnicities’ were excluded from the study because the number was too small to enable a meaningful analysis.

Demographic and clinical data such as ethnic background, family history of prostate cancer information, height, weight and urological voiding history (International prostate symptom score, IPSS) [9] was ascertained using a structured questionnaire (proforma) requiring input from patients interviews, as well as medical examinations. All men underwent digital rectal examination (DRE) and transrectal ultrasonography (TRUS) to detect abnormalities in the prostate gland and determine the prostate volume. Blood sample of each individual was collected and analysed for total PSA levels in three main centers using ADVIA Centaur PSA assay (Siemens Healthcare Diagnostic Inc, Muenchen, Germany), ARCHITECT total PSA assay (Abbott Laboratories, Abbott Park, Illinois, USA) and AxSYM total PSA assay (Abbott Laboratories, Abbott Park, Illinois, USA). The correlation between the three assays had been validated in a previous study [10].

Based on serum PSA, DRE and TRUS findings, 144 men found with PSA above 4 ng/ml were recommended for TRUS-guided prostate biopsy. Only approximately 40% (57/144) of these subjects agreed to undergo biopsy, of which 19 men (33.3%) were diagnosed with prostate cancer. With the exclusion of men with confirmed prostate cancer and those who refused the biopsy, 1054 men (90.9%) with no evidence of prostate cancer formed the study population.

### Data analysis

The relationship between baseline serum PSA level and age was tested using Spearman rank correlation. For each ethnicity, the serum PSA level was calculated for the 25^th^, 50^th^ and 75^th^ percentiles and stratified by age groups of 40–49, 50–59, 60–69 and 70–79 years. We then compared the PSA levels in our population with four other measurements performed in previous studies (Table 1). Similar approach was used to demonstrate the prostate volume and prostate density of the study population (Table 2). Subjects were then categorized into two groups using the age-specific median PSA level as a cutoff value. Of note, men with a serum PSA level above the age specific median level had a higher risk for prostate cancer compared to those of PSA below this level [11]–[13]. Potential variables associated with serum PSA level were compared between these two groups, including ethnicity, family history of prostate cancer, height, weight, the presence of LUTS, DRE (presence or absence of a nodule) and prostate volume. Logistic regression was employed to study the associations of these variables, alone and in combination, with the age-specific PSA level. Approximately 25% of the men had missing values on one or more covariates which were likely to be missing at random. Missing values were imputed using multiple imputation [14] in SPSS for Windows version 21.0 (SPSS Inc., Chicago, Illinois, USA). All variables of the multivariable logistic regression model were included in the imputation model and 10 imputation sets were created. Two-tailed *P* value <0.05 was termed as statistically significant.

**Table 1:** The median serum PSA concentration of the Asian and Western population across different age groups.

	PSA (ng/ml)						
	(25^th^ percentile; 75^th^ percentile)						
Age group (year)	Caucasian [16]	Japanese [17]	Korean [20]	Chinese (China) [19]	Chinese (Malaysia)	Malay (Malaysia)	Indian (Malaysia)
40–49	0.7	0.6	0.8	0.5	1.0	0.6	0.6
	(0.5; 1.1)	(0.4; 0.8)	(0.6; 1.1)	(−; 1.2)†	(0.7; 1.3)	(0.4; 0.9)	(0.2; 2.4)
50–59	1.0	0.7	0.9	0.8	1.0	1.0	0.8
	(0.6; 1.4)	(0.5; 1.2)	(0.6; 1.3)	(−; 2.4)†	(0.6; 1.5)	(0.6; 1.7)	(0.5; 1.3)
60–69	1.4	0.9	1.0	0.9	1.4	1.2	0.9
	(0.9; 3)	(0.5; 1.5)	(0.7; 1.6)	(−; 3.2)†	(0.8; 2.2)	(0.7; 1.9)	(0.6; 1.3)
70–79	2.0	1.4	1.3	1.2	1.6	1.4	1.4
	(0.9; 3.2)	(0.7; 2.1)	(0.9; 2.5)	(−; 3.4)†	(0.9; 2.6)	(1.0; 2.7)	(0.6; 2.3)
**Total subjects**	471	286	5801	830	526	378	150

† Only 95^th^ percentile was documented in [19].

**Table 2:** The comparison of prostate volume and PSA density between the Asian and Western population across various age groups.

	Prostate volume (ml)					PSA density (ng/ml^2^)				
	(25^th^ percentile; 75^th^ percentile)					(25^th^ percentile; 75^th^ percentile)				
Age group(year)	Caucasian [16]	Japanese [17]	Chinese(Malaysia)	Malay(Malaysia)	Indian (Malaysia)	Caucasian [16]	Japanese [17]	Chinese (Malaysia)	Malay (Malaysia)	Indian (Malaysia)
40–49	23.5	16.8	25.5	22.3	18.7	0.03	0.04	0.04	0.03	0.03
	(20.4; 29.0)	(14.5; 18.5)	(18.0; 28.9)	(18.0; 29.1)	(17.0; 20.9)	(0.02; 0.04)	(0.03; 0.05)	(0.03; 0.07)	(0.02; 0.04)	(0.01; 0.06)
50–59	30.7	17.4	24.0	25.5	26.0	0.03	0.04	0.04	0.04	0.03
	(23.0; 37.1)	(15.4; 22.2)	(19.0; 30.9)	(20.7; 33.4)	(20.4; 31.3)	(0.02; 0.05)	(0.03; 0.07)	(0.03; 0.06)	(0.02 0.06)	(0.02; 0.06)
60–69	34.6	18.5	28.5	28.5	26.0	0.05	0.05	0.05	0.04	0.04
	(28.0; 43.7)	(15.7; 21.2)	(21.5; 40.0)	(21.0; 36.7)	(21.1; 34.6)	(0.03; 07)	(0.03; 0.07)	(0.03; 0.08)	(0.03; 0.07)	(0.02; 0.05)
70–79	35.4	19.1	29.1	35.0	25.3	0.05	0.07	0.05	0.05	0.04
	(29.6; 51.4)	(15.8; 25.1)	(24.0; 36.3)	(22.0; 39.5)	(21.6; 31.0)	(0.03; 0.08)	(0.04; 0.09)	(0.04; 0.07)	(0.03; 0.07)	(0.03; 0.09)
**Total subjects**	471	286	526	378	150	471	286	526	378	150

## Results

A total of 1054 participants were enrolled in the study with an average age of 58.99±7.32 years; of which 73 (6.9%) aged between 40–49 years, 492 (46.7%) were between 50–59 years 395 (37.5%) ranged between 60–69 years and 94 (8.9%) varied between 70–79 years. Most of the subjects were Chinese (526; 49.9%), followed by Malays (378; 35.9%) and Indians (150; 14.2%). Twenty-seven (2.6%) subjects had a positive family history of prostate cancer. The overall median PSA level was 1.04 ng/ml (range 0.09–16.47 ng/ml) and it varied across different ethnicities. For instance, the median PSA level of Malay, Chinese and Indian was 1.00 ng/ml, 1.16 ng/ml and 0.83 ng/ml respectively (*p* = 0.000; Kruskal Wallis test). Overall, 38 (3.6%) men had a serum PSA level above the normal threshold (4 ng/ml) and no clinical evidence of prostate cancer.

There was a positive trend between serum PSA level and age (Table 1), achieving a formal statistical significance (*r* = 0.247; *p* = 0.000, Spearman rank correlation). Comparing the 10-year age groups, Indian had a relatively lower median PSA level than Malay and Chinese who shared a comparable median PSA value across all age groups (Table 1). This trend was also consistently observed in prostate volume in which Chinese and Malays had a higher prostate volume than the Indians (Table 2). Although the median PSA value and prostate volume of Japanese were fairly lower compared to our study population, both populations showed a similar value of the median PSA density across all age groups (Tables 1 and 2). In addition, Caucasian tends to have a relatively higher median PSA level and prostate volume, but a lower PSA density, than Asians (Tables 1 and 2).

Subjects were then grouped into two categories using the age-group specific median PSA level as cut-off point. Summary estimates obtained after multiple imputation were similar to those in complete cases. Ethnicity, weight and prostate volume were found to be significantly associated with age-specific PSA level (Table 3). For instance, men of Indian ethnicity were 40% less likely to have an above-median age-specific PSA level than the Malays (*p* = 0.007). On the other hand, subjects with a prostate volume ≥30 ml was 3.5-fold more likely than those of a lower prostate volume to have a PSA level above the age-specific median (*p*<0.001). With every 1 kg increase in body weight, there was a 1% decline in the probability of having an above-median age-specific PSA level (*p* = 0.003). We did not find any significant associations between age-specific PSA level and family history of prostate cancer, height, LUTS or DRE. In the multivariable analysis, it was found that ethnicity, weight and prostate volume remained to be significantly and independently associated with age-specific median PSA level (Table 4).

**Table 3:** Comparison of factors associated with median PSA level.

	Frequency distribution				Pooled results		
	PSA ≤ age specific median†		PSA>age specific median†				
Factors	No.	%	No.	%	OR	95% CI	*P*
**Ethnicity**							
Malay	195	36.7	183	35.0	1.00		
Chinese	240	45.1	286	54.8	1.27	0.97–1.66	0.077
Indian	97	18.2	53	10.2	0.58	0.39–0.86	*0.007*
**Family history of PC**							
Absent	514	96.6	513	98.3	1.00		
Present	18	3.4	9	1.7	0.50	0.22–1.13	0.094
**Height (m)** *	531	50.6	518	49.4	1.01	1.00–1.03	0.318
Mean	165.25		165.71				
**Weight (kg)** *	531	50.6	519	49.4	0.99	0.98–1.00	*0.003*
Mean	71.78		69.45				
**LUTS (IPSS score)** *							
≤7	270	52.0	241	47.8	1.00		
>7	249	48.0	263	52.2	1.18	0.93–1.51	0.180
**DRE** *							
No nodule	478	92.5	476	93.7	1.00		
Nodule	39	7.5	32	6.3	0.83	0.51–1.34	0.439
**Prostate volume (ml)** *							
<30	318	79.7	187	48.1	1.00		
≥30	81	20.3	202	51.9	3.48	2.55–4.75	*0.000*

† Based on the study population, the age-specific median PSA level was defined as 0.69 ng/ml, 0.93 ng/ml, 1.21 ng/ml and 1.52 ng/ml for the age groups of 40–49 years, 50–59 years, 60–69 years and 70–79 years, respectively. Abbreviations: CI, confidence interval; DRE, digital rectal examination; IPSS, international prostate symptom score; LUTS, lower urinary tract voding symptoms; OR, odds ratio; PC, prostate cancer.

*Percentage of subjects with missing values on height (0.5%), weight (0.4%), LUTS (2.9%), DRE (2.8%) and prostate volume (25.2%).

**Table 4:** Multivariable analysis of factors associated with age-specific median PSA level.

Factors	Regression coefficient*	OR	95% CI	*P*
**Ethnicity**				
Malay	Baseline	1.00		
Chinese	0.187	1.21	0.90–1.61	0.207
Indian	−0.489	0.61	0.40–0.93	*0.022*
**Weight**	−0.020†	0.98	0.97–1.00	*0.001*
**Prostate volume**				
<30	Baseline			
≥30	1.331	3.79	2.71–5.28	*0.000*

*Multivariate model inlcudes ethnicity, weight and prostate volume.

† Weight (kg) considered as continuous variable within the multivariate model.

Abbreviations: CI, confidence interval; OR, odds ratio.

## Discussion

Findings from this study provide an insight into the baseline PSA profile, prostate volume and PSA density of a large cohort of multiethnic Asian men. We demonstrated that there were significant ethnic variations in age-specific PSA levels, independent of body weight and prostate volume.

The elevated serum PSA concentration is often seen in men with benign prostatic hyperplasia (BPH), prostatitis or prostate cancer in some cases. Based on the current study, the detection rate of prostate cancer amongst men with PSA>4 ng/ml who underwent TRUS-guided biopsy was 1.8% only (19/1054), raising a concern of the existing PSA threshold that is based on Caucasian populations. Firstly, the prostate cancer incidence in Asia has increased in the last two decades with a higher proportion of patients being diagnosed with high grade tumor in comparison to the Caucasians [15]. Secondly, evidence from previous studies revealed that Asian tends to have a higher PSA density but a lower level of PSA and prostate volume, in comparison to Caucasians (Tables 1 and 2) [16], [17]. Therefore, it is essential to understand the baseline PSA profile and its associated factors in Asian populations. Notably, the baseline PSA level is a strong indicator for predicting subsequent clinically diagnosed prostate cancer, raising the possibility of risk stratification in prostate cancer screening [11].

We demonstrated that age and ethnicity were amongst the significant variables associated with baseline median PSA level. Besides the significant association of serum PSA concentration with age (*p* = 0.000), our results showed that Indians were more likely to have a lower median PSA than Malay (*p* = 0.007; univariable analysis) whilst a comparable PSA level was described amongst Malay and Chinese (*p* = 0.077; univariable analysis) after adjusting for age-group. The age-dependent PSA trend was consistently observed in studies involving either Caucasian [16] or Asian [17]–[20] across all age groups. For instance, Osterling *et al* [17] found a direct correlation between the age of the Japanese men and the PSA level (correlation coefficient, *r* = 0.33, *p*<0.001); the serum PSA level of the Japanese was lower than the whites even after adjusting for age (*p*<0.001). Interestingly, results from our study demonstrated that the median PSA level of our population, except Indian, was relatively higher compared to other Asian studies but similar to the Caucasian (Table 1). These discrepancies could be due to methodological differences between the current and four previous reports [16], [17], [19] and [20], although the fundamental differences in the biology of the PSA in various ethnicities remain under investigation. For example, subjects with history of prostate cancer, prostatectomy or other specified conditions that would interfere with voiding function were excluded from the previous studies [16], [17], [19], [20]. Conversely, subjects who did not undergo 5α-reductase inhibitor treatment for known or newly diagnosed LUTS (IPSS>7) were included in the current study, suggesting a potential skew towards higher baseline PSA levels. Although the serum PSA level was measured with different PSA assays in the five studies, there was an excellent correlation of these total PSA assays based on the results from earlier studies [10], [19]–[22]. For instance, the serum PSA concentration measured using the IMx instrument in [17] demonstrated a high correlation with those reported with Tandem-R (*r* = 0.99) [22], ELSA-PSA (*p*<0.001) [19] and AxSYM assays (*r* = 0.99) [21]. Of note, the latter three assays were used in [16], [19] and current studies respectively. Similar trend was observed in the comparisons of assays performed in the Caucasian [16] and Korean [20] studies with a strong correlation of *r* = 0.98 and *p* = 0.0001 [20]. In this study, the *r^2^* values were >0.983 when comparing ARCHITECT with ADVIA Centaur and AxSYM total PSA assays [10]. It is noteworthy that the presence of interassay variability may be resulted from the different epitope specificity of the antibodies used [23].

Our finding revealed that the PSA level was significantly associated with prostate volume, in parallel with previous studies [24]–[32]. Being the most significant predictor of PSA levels with an odds ratio of 3.79 in the multivariable analysis, it is suggested that prostate volume could be used as a tool for estimating the degree of prostate enlargement accurately, and for therapeutic decision-making [33]. Notably, the prostatic cellular composition differs between Asians and Whites in which the prostates of Chinese have significantly more glandular lumens but less connective tissue and smooth muscle than Caucasian [34]. We also observe significant inverse association between PSA level and body weight in the multivariable analysis. It is hypothesized that the lower PSA level associated with obesity is due to PSA haemodilution, a condition in which the total amount of PSA in the blood (PSA mass) is unaffected by an increased blood volume in obese man [31], [32]. Other factors including difficulties in performing a thorough DRE and larger prostates might lead to the detection bias and fewer early stage of prostate cancer diagnosed in obese individuals [35], [36].

A positive trend was demonstrated between the presence of LUTS (IPSS score >7) and the baseline serum PSA concentration albeit not achieving statistical significance (*p* = 0.180; univariable analysis). Subjects with LUTS may have an elevated PSA level resulting from BPH-related prostatic enlargement or prostatic inflammation; nevertheless, there are several other causative factors of LUTS including the aging of the bladder muscle, a dynamic component of the smooth muscle tone of the prostate, metabolic factors and serum sex steroid [37]–[40]. Results from a recent Korean study demonstrated that IPSS did not improve the predictive value of PSA in spite of their significant correlation [30]. There was no clear association between DRE and the PSA level (Table 3). Nevertheless, the presence of nodule during DRE was significantly and positively associated with diagnosis of prostate cancer (data not shown).

Our study findings provide evidence of the PSA profiles and their associated factors within a multiethnic Asian population. An important clinical implication of this study is that ethnicity may also need to be taken into account when investigating serum PSA concentrations [41] for prostate cancer detection in a multi-ethnic Asian population, in addition to age and prostate volume.
